# Lactylation in digestive system tumors: from mechanisms to therapeutic target

**DOI:** 10.3389/fonc.2025.1607249

**Published:** 2025-12-11

**Authors:** Jun Wei, Qian Ding, Hongjun Wang, Yang Liu

**Affiliations:** 1 School of Basic Medical Sciences, Jilin Medical University, Jilin, China; 2 Edinburgh Medical School: Biomedical Sciences, College of Medicine and Veterinary Medicine, The University of Edinburgh, Edinburgh, United Kingdom; 3 Zhejiang University-University of Edinburgh Institute, Zhejiang University School of Medicine, Haining, China; 4 Shanghai Fourth People’s Hospital, School of Medicine, Tongji University, Shanghai, China

**Keywords:** digestive system tumors, epigenetic modification, lactylation, therapeutic target, tumor metabolism

## Abstract

Lactylation, a recently identified epigenetic modification derived from lactate metabolism, has emerged as a key regulator linking cellular metabolic states to chromatin remodeling and gene transcription. Acting through histone and non-histone protein lactylation (for example, Histone H3 Lysine 9 Lactylation [H3K9la], Histone H3 Lysine 18 Lactylation [H3K18la]), this modification reshapes chromatin accessibility and activates transcriptional programs, thereby driving tumor progression, metabolic reprogramming, immune evasion, and chemoresistance in digestive system malignancies. This review comprehensively summarizes the latest advances in lactylation across esophageal cancer (EC), gastric cancer (GC), colorectal cancer (CRC), hepatocellular carcinoma (HCC), pancreatic cancer (PC), and gallbladder cancer (GBC), emphasizing its role in epigenetic regulation of oncogenic signaling and metabolic–epigenetic crosstalk. Moreover, we discuss potential biomarkers, therapeutic targets, and pharmacologic strategies aimed at modulating lactylation. Despite promising translational potential, key challenges remain in standardizing detection methods and validating clinical efficacy. The intricate mechanisms of lactylation not only deepen our understanding of digestive tumor biology but also unveil a rich landscape of novel therapeutic targets. Future investigations should focus on deciphering lactylation-mediated epigenetic mechanisms in tumor immunotherapy and precision medicine, providing new directions for research and clinical insights for the early diagnosis and tailored treatment of digestive system tumors.

## 1 Introduction

Digestive system malignancies, including esophageal cancer (EC), gastric cancer (GC), colorectal cancer (CRC), hepatocellular carcinoma (HCC), pancreatic cancer (PC), and gallbladder cancer (GBC), collectively account for more than 30% of global cancer cases and approximately 35% of cancer-related deaths according to GLOBOCAN 2020 data (1). These cancers therefore represent a major global health challenge. Marked geographic and demographic variations exist: the highest incidence and mortality rates are observed in East Asia (particularly China, Japan, and Korea), as well as in parts of Eastern Europe and South America (2). Helicobacter pylori infection remains a key etiological factor for GC, especially in East Asian populations, while CRC incidence continues to rise in newly industrialized countries due to dietary westernization and sedentary lifestyles (3). HCC is endemic in regions with high hepatitis B and C virus prevalence, such as East Asia and sub-Saharan Africa (4). PC shows increasing incidence worldwide, particularly in developed countries, and GBC exhibits distinct regional clustering in Chile, northern India, and Japan (5).

In the energy metabolic pathways of cells, glycolysis and oxidative phosphorylation (OXPHOS) serve as the “powerhouses” of the cell, supplying the necessary energy for various cellular activities to maintain normal cellular operations while generating a plethora of tightly coupled intermediate metabolites (6). These metabolites participate in numerous biochemical reactions within the cell, acting as key nodes in the cellular metabolic network (7). For a long time, lactate was considered a mere metabolic waste; however, in cancer research, it has garnered attention as a significant product of glycolysis due to the Warburg effect associated with aerobic glycolysis (6). Notably, cancer cells produce substantial amounts of lactate and adenosine triphosphate (ATP) through glycolysis, even under fully aerobic conditions, a phenomenon known as the Warburg effect (8, 9). This effect significantly increases lactate production, thereby acidifying the tumor microenvironment (TME) (10). The acidic TME fosters conditions conducive to the growth and survival of cancer cells, facilitating their continuous proliferation (11). Research findings from the past three years have further elucidated that lactate exhibits remarkable metabolic and non-metabolic functions (12). Recently, a significant breakthrough was achieved by Di Zhang et al., who confirmed for the first time that lactate-induced lactylation constitutes a novel epigenetic modification (13).

Beyond the Warburg effect, understanding the biochemical formation and transport of lactate is essential for clarifying its biological significance in tumors. Under normal aerobic conditions, pyruvate generated from glycolysis enters the mitochondria and is oxidatively decarboxylated by the enzyme pyruvate dehydrogenase (PDH) to produce acetyl-CoA, which then fuels the tricarboxylic acid (TCA) cycle for efficient ATP production (6, 14). However, in tumor cells or under hypoxic stress, the activity of PDH is often suppressed through inhibitory phosphorylation by pyruvate dehydrogenase kinases (PDKs), diverting pyruvate toward lactate dehydrogenase A (LDHA)-mediated conversion into lactate (15, 16). Meanwhile, lactate dehydrogenase B (LDHB) catalyzes the reverse reaction in oxidative cells, converting lactate back to pyruvate for energy metabolism (17, 18). This enzymatic balance establishes a dynamic lactate–pyruvate axis that reflects cellular metabolic plasticity.

Once synthesized, lactate is transported across cellular membranes by monocarboxylate transporters (MCTs), primarily Monocarboxylate Transporter 1 (MCT1, SLC16A1) and Monocarboxylate Transporter 4 (MCT4, SLC16A3) (19). MCT4 facilitates lactate efflux from highly glycolytic tumor cells, while MCT1 enables lactate uptake into oxidative or stromal cells (20). This bidirectional transport forms an intercellular “lactate shuttle” that redistributes energy substrates and signaling molecules within the tumor microenvironment. The accumulation and circulation of lactate through this shuttle system not only contribute to the acidification and immunosuppressive remodeling of the tumor microenvironment (21) but also create a metabolic context that enables lactate-derived epigenetic modifications, such as lactylation (22).

Histones, unique compounds in chromatin composed of nucleosome core proteins and DNA, undergo various post-translational modifications (PTMs) that play a crucial regulatory role in gene expression. Classic PTMs, such as acetylation, malonylation, and succinylation, regulate numerous processes in cellular physiology and biochemistry by altering the spatial structure of proteins (23). In contrast, histone lactylation represents a novel form of PTM. In M1 macrophages, lactate functions as a signaling molecule that stimulates gene transcription through histone lysine lactylation, thereby playing a unique role in the regulation of cellular gene expression (24).

**Figure 1:**
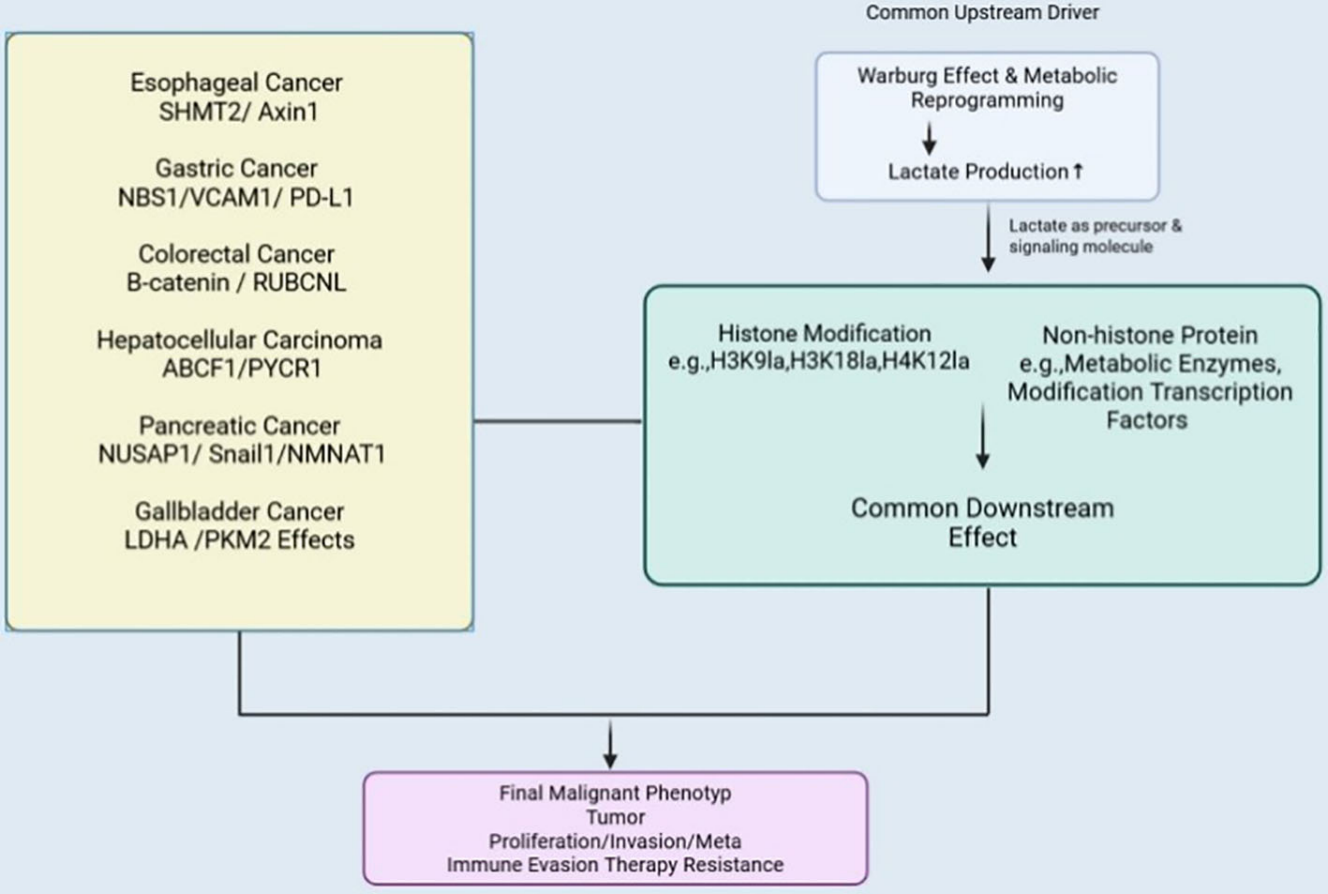
Flowchart summarizing lactylation mechanisms across different cancer types.

The selection of these six cancer types in this review is based on their shared metabolic reprogramming characteristics, particularly enhanced glycolysis and lactate accumulation, which are closely associated with lactylation-dependent epigenetic regulation. Elucidating the mechanisms of lactylation across these major digestive system tumors provides a unified framework for understanding tumor progression, immune evasion, and therapeutic resistance, and may facilitate the discovery of novel biomarkers and therapeutic targets. To provide a comprehensive overview of the role of lactylation across these cancers, a flowchart summarizing the common mechanisms and their impact on tumor progression is presented below (**Figure 1**).

This flowchart illustrates the upstream drivers (e.g., Warburg effect, metabolic reprogramming) leading to lactate production, which promotes lactylation. Lactylation, involved in both histone and non-histone protein modifications, contributes to effects such as tumor proliferation, immune evasion, and chemotherapy resistance. The chart synthesizes lactylation’s role across different digestive system cancers, highlighting its impact on key pathways in each cancer type.

## 2 Core mechanism of lactylation

Lactylation is a reversible, lactate-mediated post-translational modification of lysine. By modifying both histones and non-histone proteins, it plays a pivotal role in gene transcription, signal transduction, and metabolic regulation. This mechanism is particularly crucial in linking metabolic abnormalities (such as the Warburg effect in tumors) with pathological processes of diseases (especially cancer), including drug resistance and immune microenvironment remodeling (25).

**Figure 2:**
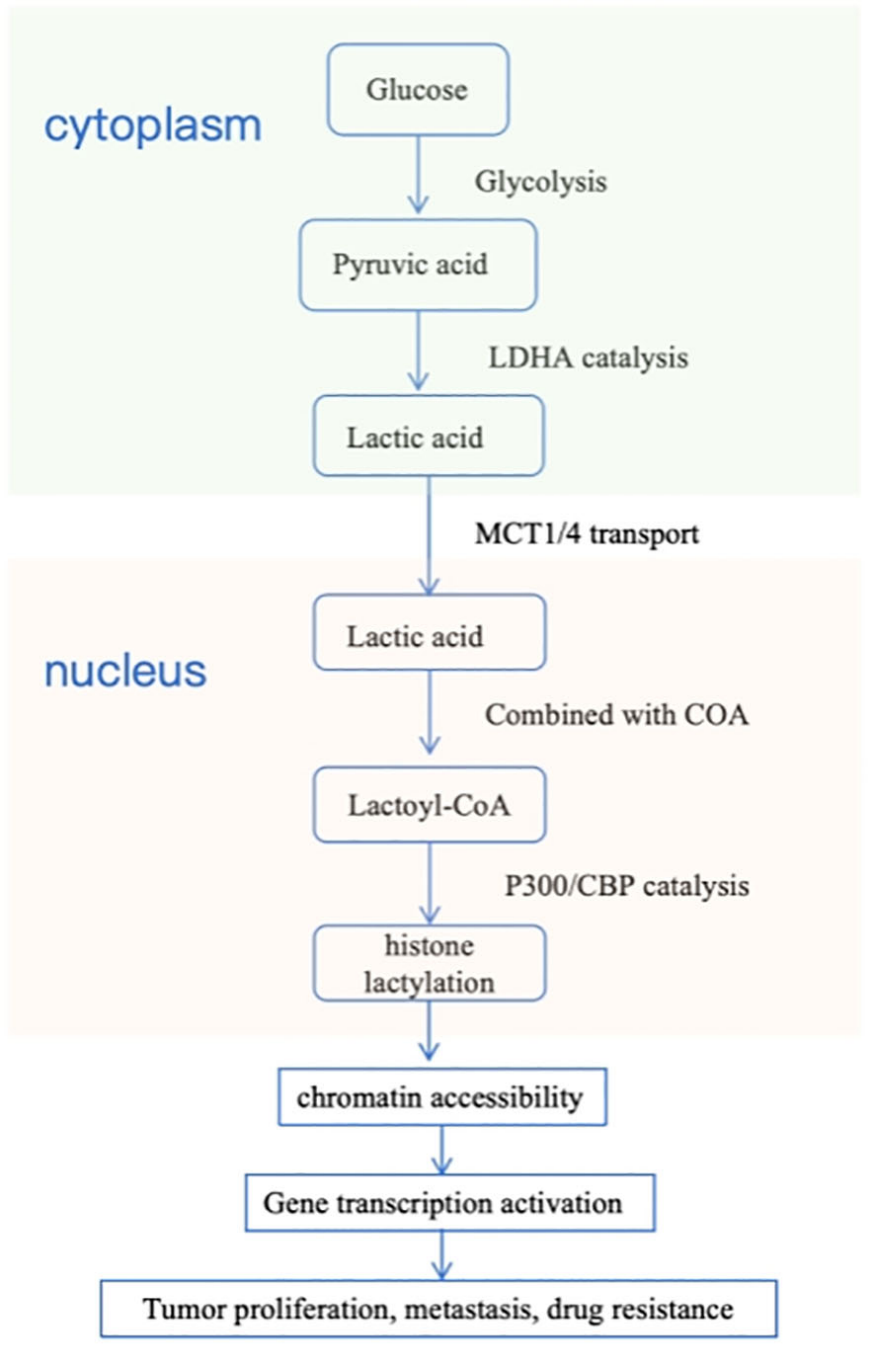
The core mechanism of lactylation modification.

The core regulatory mechanism of protein lactylation relies on a sophisticated molecular system composed of “writers,” “erasers,” and “readers.” In this mechanism, the metabolite lactate is first activated by “writers” into lactyl-CoA or directly serves as a substrate, subsequently covalently modifying specific lysine residues on histone and non-histone substrates to form protein lactylation modifications. This modification signal can be recognized by specific “reader” proteins and translated into precise regulation of cellular processes such as proliferation and differentiation through modulation of downstream signaling pathways or chromatin states. To terminate the signal, “erasers” efficiently remove the lactyl groups from lysine residues, resetting the modification. Thus, this complete “write-erase-read” dynamic cycle tightly couples the metabolic state (lactate level) of cells with gene expression and cellular functions, forming a crucial metabolic-epigenetic regulatory axis that plays a pivotal role in cell fate determination and disease development (26). Moreover, studies have revealed three key source pathways of lactylation. Firstly, L-lactyl-CoA serves as the primary precursor for enzymatic L-lactylation, catalyzed by acetyl-CoA synthetase 2 from L-lactate in both nuclear and cytoplasmic compartments. Although its abundance is significantly lower than that of acetyl-CoA, it is sufficient to drive lactylation modifications of histones and non-histone proteins under glycolytically active conditions. Secondly, S-D-lactylglutathione functions as the direct donor for non-enzymatic D-lactylation, derived from the methylglyoxal detoxification pathway, the glyoxalase system. This pathway operates independently of glycolytic flux, and its accumulation can markedly enhance D-lactylation levels. Third, L-lactyl-AMP represents a newly discovered enzymatic pathway. Under high-lactate conditions, alanyl-tRNA synthetase can catalyze the formation of this intermediate, which subsequently transfers the lactyl group to target proteins, revealing a CoA-independent lactylation mechanism. These multi-layered lactyl supply networks precisely regulate the extent and functional specificity of lactylation from the metabolic source, establishing its pivotal role in linking cellular metabolism with signal transduction (27) (**Figure 2**).

Lactylation modification directly links cellular metabolism (glycolysis) with epigenetic regulation, which is coordinately accomplished by three classes of enzymes: lactate-producing enzymes (LDHA), lactate-transporting enzymes (MCTs), and lactylation-modifying enzymes (p300/CBP).

## 3 The mechanism of lactylation modification in digestive system tumors

### 3.1 Esophageal cancer

In the development of EC, lactate metabolism and its associated mechanisms significantly promote tumor progression and deterioration by creating an acidic TME. The overexpression of LDHA leads to increased production and accumulation of lactate, which provides energy and carbon sources for the rapid growth of cancer cells. This process promotes the acidification of the TME, enhances angiogenesis, and impairs the anti-tumor functions of immune cells (28). These metabolic alterations are closely associated with poor prognosis in patients with EC (29).

Metabolomic studies have further revealed significant metabolic differences between EC tissues and non-tumor tissues. Research indicates that the concentrations of lactate and citrate are significantly elevated in EC tissues, reflecting the presence of the “Warburg effect.” Additionally, the levels of amino acids, particularly glutamine, in tumor tissues are significantly elevated, which may be related to the excessive degradation and utilization of glutamine during tumor development (30). At the genetic level, studies have shown that silencing Caprin-1 can significantly inhibit the proliferation and glycolytic activity of EC cells while reducing the expression of METTL3 and WTAP, thereby suppressing tumor growth (31). Similarly, inhibiting MCTs can block lactate transport, significantly increasing the apoptosis rate of cancer cells and inhibiting their growth capacity (32).

Lactylation, as a key mechanism, promotes the development of EC through multiple pathways. As a histone mark (for example, H3K9la), lactylation directly reshapes chromatin accessibility and modulates the transcriptional activity of tumor-associated genes (33). For example, in a hypoxic environment, lactate accumulation induces SHMT2 protein lactylation, enhancing its enzymatic activity, promoting glycolytic metabolism and energy supply, while maintaining cancer stem cell characteristics such as self-renewal and chemotherapy resistance (34). Recent studies have further revealed that lactylation-driven stemness in EC is closely associated with metabolic–epigenetic crosstalk. Specifically, SHMT2 lactylation enhances one-carbon metabolism and supports nucleotide synthesis, thereby sustaining the proliferative capacity of cancer stem-like cells (34). Meanwhile, lactate-induced histone lactylation activates the transcription of key stemness regulators, including c-Myc and SOX2, through chromatin remodeling at their promoter regions. Moreover, activation of the Wnt/β-catenin and Nuclear Factor kappa-light-chain-enhancer of activated B cells(NF-κB) signaling pathways has been observed in lactate-enriched microenvironments, linking lactylation-dependent transcriptional activation to the maintenance of stem cell phenotypes and chemoresistance in esophageal squamous cell carcinoma (35). These findings suggest that lactylation contributes to cancer stemness both by reprogramming cellular metabolism and by epigenetically enhancing oncogenic transcriptional networks. Additionally, lactylation modification of histone H3K9 (H3K9la) acts as an activating epigenetic mark, facilitating the recruitment of transcriptional coactivators to the LAMC2 promoter and thereby enhancing the proliferation and migration capabilities of esophageal squamous cell carcinoma (33). Lactylation also inhibits the degradation of Axin1, strengthening the glycolytic pathway and providing continuous energy support for EC cells (33). Meanwhile, the long non-coding RNA AP001885.4 promotes the expression of c-myc by inducing histone lactylation and NF-κB (p65)-dependent transcriptional activation, enhancing its mRNA stability through METTL3, and ultimately facilitating the proliferation of esophageal squamous cell carcinoma (36).

In summary, lactylation plays a pivotal role in the metabolic reprogramming, epigenetic regulation, and maintenance of stemness in EC (22, 37). Through both histone and non-histone protein modifications, lactate reshapes transcriptional and metabolic networks that drive tumor aggressiveness (38). Specifically, histone lactylation, mainly occurring on H3K9, H3K18, and H4K12 residues, enhances chromatin accessibility and activates oncogenic gene expression programs. For instance, H3K9la at the LAMC2 promoter facilitates tumor cell migration and invasion (33), while H3K18la-mediated activation of c-Myc and SOX2 promotes proliferation and stemness maintenance. In addition, non-histone lactylation of SHMT2 and Axin1 strengthens glycolytic flux and confers chemoresistance by sustaining energy production and redox balance (34, 39). Collectively, these findings suggest that lactylation promotes the proliferation, migration, and therapeutic resistance of EC cells by integrating metabolic remodeling with epigenetic activation of oncogenic pathways.

### 3.2 Gastric cancer

In recent times, investigations into the mechanisms of GC development have increasingly highlighted the crucial function of tumor metabolic reprogramming in both its initiation and advancement. Metabolomic analyses have demonstrated a notable rise in glycolytic intermediates, including lactate and pyruvate, within GC tissues compared to normal tissues (40). At the same time, inositol metabolism related to fatty acid synthesis in GC tissues shows marked activation, reflecting a simultaneous boost in glycolysis and lipid synthesis within the metabolic landscape of GC. These metabolic alterations not only provide the energy and biosynthetic precursors required for the rapid proliferation of GC cells but also render them metabolically addicted to glycolysis. This dependency makes gastric tumor cells particularly sensitive to glycolytic inhibitors such as 2-deoxy-D-glucose (2DG), since blocking this dominant energy producing pathway leads to a greater impairment of survival in tumor cells than in normal cells (41). Research conducted by Maruyama et al. indicated that glycolysis inhibitors, like 2-DG, significantly impede the proliferation of GC cells and increase their vulnerability to chemotherapy, suggesting that glycolysis could be a viable target for therapeutic intervention (42).

In this context, lactylation, as an emerging epigenetic modification mechanism, is considered a crucial regulatory factor in the progression of GC. Utilizing bioinformatics methods, Yang et al. discovered that four metabolic pathways closely related to lactylation were significantly overexpressed in GC tissues. Furthermore, the lactylation score was confirmed to be closely associated with the overall survival and disease progression of GC patients. Patients with higher lactylation scores not only exhibited more extensive immune cell infiltration, particularly a significant increase in macrophage infiltration, but also displayed higher genetic instability (43). These characteristics further suggest that tumors with elevated levels of lactylation may possess stronger immune evasion capabilities and a higher risk of functional disruption. Studies have found that immune checkpoint inhibitors (ICIs) exhibit reduced efficacy in GC patients with higher lactylation scores. This indicates that the level of lactylation may exert an inhibitory effect on the effectiveness of immunotherapy (43). Currently, there is no universally accepted or validated criterion for lactylation scoring in GC. Most existing studies define lactylation levels based on quantitative immunohistochemistry or proteomic analysis of histone lactylation marks such as H3K18la and H3K9la (44). “High-lactylation” tumors are generally identified as those with histone lactylation signal intensities above the cohort median or based on bioinformatics-derived clustering models. However, these approaches rely on limited patient samples and remain exploratory in nature (45). The observed association between elevated lactylation and reduced immunotherapy efficacy should be interpreted cautiously, as further validation in larger, clinically annotated cohorts is required (46). However, reducing lactylation levels to enhance tumor sensitivity to ICIs could represent a promising therapeutic strategy.

Mass spectrometry analysis additionally confirmed the involvement of lactylation in GC. A comprehensive examination of AGS cells in GC revealed a total of 2,375 lysine lactylation sites across 1,014 distinct proteins, many of which play a role in the modulation of spliceosome activity. Notably, levels of lactylation were significantly heightened in GC tissues, and an association was found between increased lactylation levels and poor patient prognosis. These investigations not only enriched the database of lactylated proteomes specific to GC but also reinforced the potential of lactylation as a prognostic marker (47). Facing difficulties in accurately pinpointing lactylation sites through mass spectrometry, Lai et al. introduced Auto-Kla, a tool based on automated machine learning designed to predict protein lactylation locations. This instrument is anticipated to act as a valuable asset for identifying post-translational modification sites and aiding in the creation of related predictive models (48). In conclusion, lactylation functions as a crucial regulatory element in the metabolic reprogramming of GC, exhibiting a close relationship with patient prognosis, immune microenvironment, and responses to treatment. Future research could explore the possibility of improving GC treatments through the modulation of lactylation, thus providing a foundation for the development of novel therapeutic strategies.

### 3.3 Colorectal cancer

**Figure 3:**
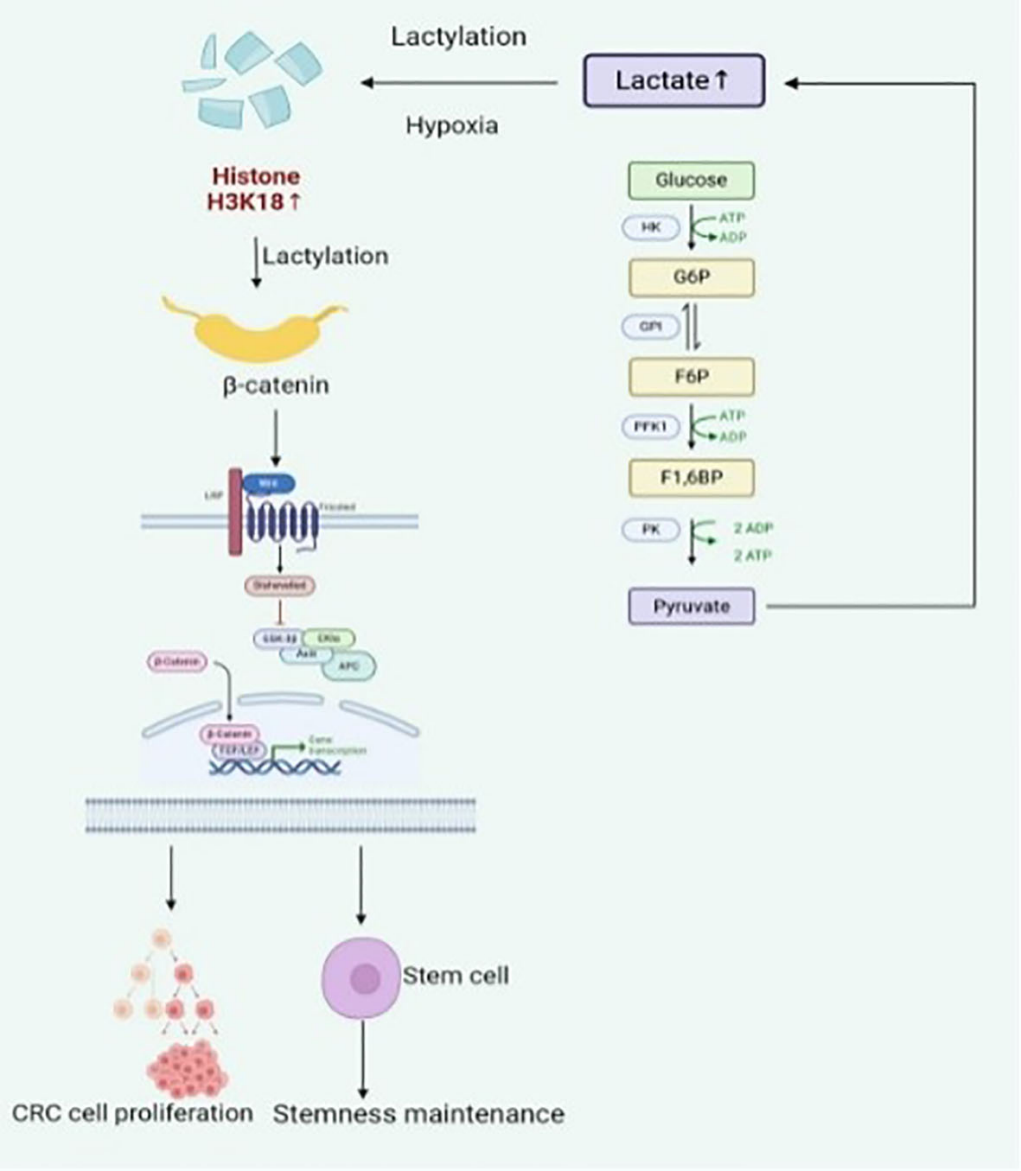
The core role of lactylation in colorectal cancer.

CRC stands as one of the primary contributors to global cancer-related deaths and disease burden, no doubt. A critical epigenetic mechanism, lactylation, has been demonstrated to influence CRC development and advancement substantially. As a histone post-translational modification (notably at lysine residues such as H3K18la and H4K12la) (49), lactylation reshapes chromatin accessibility and transcriptional activity (37), thereby linking metabolic state to epigenetic gene regulation in CRC (45). Observed in studies is that glycolysis becomes markedly upregulated in CRC cells under hypoxia, with lactate production consequently rising (50). Lactate acts as a substrate for lactylation, leading to elevated levels of histone H3K18 lactylation, a phenomenon closely correlated with enhanced tumor cell invasiveness (51). Examples include β-catenin undergoing lactylation in low-oxygen environments (50), resulting in Wnt signaling pathway activation and subsequent promotion of CRC cell proliferation along with stemness maintenance (50). These observations imply metabolic reprogramming and signaling cascades being regulated by lactylation, thereby driving oncogenesis forward. This evidence underscores a metabolic–epigenetic crosstalk, wherein lactylation-mediated chromatin remodeling enhances β-catenin transcriptional activity and sustains Wnt signaling in CRC (41, 52). Also noted is chemotherapy resistance in CRC cells showing strong associations with heightened lactylation activity (**Figure 3**). Tumors that produce lactate, for example, increase RUBCNL expression through H3K18 lactylation modification. The elevated presence of this autophagy-related protein then facilitates autophagy induction and contributes to bevacizumab resistance in CRC cells. Supporting this are clinical data revealing CRC patients exhibiting elevated histone lactylation levels more frequently displaying poor responses to bevacizumab therapy. Conversely, suppression of histone lactylation leads to notable reductions in tumor growth rates, survival capacity, and disease progression under hypoxic conditions (51). Mechanistically, H3K18la functions as an activating epigenetic mark, facilitating open chromatin conformation and transcriptional activation of oncogenic targets involved in invasion and metastasis (53). Novel therapeutic strategies targeting lactylation could thus emerge for overcoming chemoresistance challenges in CRC treatment approaches.

The central role of lactylation in CRC. The hypoxic tumor microenvironment upregulates glycolysis, resulting in the production of large amounts of lactate. Lactate, as a substrate, drives the lactylation modification of key proteins such as histone H3K18 and β-catenin, thereby activating oncogenic signaling, maintaining stem cell characteristics, enhancing proliferation.

The metabolic reprogramming phenomenon in CRC exhibits a strong linkage with lactylation processes. Central to the Warburg effect regulation in CRC was found by Lin et al. to be the ALDOB enzyme, facilitating both lactate production and its subsequent release. Through this mechanism activated becomes PDK1, influencing thereby the carcinoembryonic antigen (CEA) concentrations. Not merely enhancing CRC cell invasiveness does lactate demonstrate, but also profoundly altering it does the tumor microenvironment by upregulating LDHB expression in adjacent cells. Of particular importance emerges carcinoembryonic antigen-related cell adhesion molecule 6 (CEACAM6), playing a pivotal role in modulating both CRC cell proliferation and resistance to chemotherapy drugs (54). These observations collectively highlight the complex interplay existing between metabolic reprogramming events and lactylation modifications, revealing that lactylation acts as an epigenetic interpreter of metabolic flux, converting lactate accumulation into stable transcriptional changes that favor tumor adaptation and resistance. Thus it can be seen how multifaceted are the interactions wherein metabolic changes influence lactylation dynamics, while reciprocal effects manifest through tumor progression markers and treatment responses (13). Examples include the dual functionality of lactate as both metabolic byproduct and signaling molecule, alongside CEACAM6’s involvement in multiple oncogenic pathways.

By mediating histone-based epigenetic regulation, lactylation provides a molecular bridge between metabolic reprogramming and transcriptional reprogramming in CRC. Targeting this lactate–epigenetic axis holds great potential for improving therapeutic efficacy and overcoming chemoresistance (45, 55). Through histone modifications-process, metabolic reprogramming-effects, and signaling pathways-activation, enhanced are tumor invasiveness-capacity and chemotherapy resistance-properties by this modification-type. Examining the molecular mechanisms-details behind lactylation-phenomenon could provide insights into CRC pathological processes-characteristics. Such understanding-basis may serve as a foundation-solid for novel therapeutic approaches-development. Future treatment strategies-focus targeting lactylation-regulation and related metabolic pathways-components might substantially improve outcomes-treatment and prognosis-levels for CRC patients-group. Examples show this approach-direction holds considerable promise-potential in clinical applications-field.

### 3.4 Hepatocellular carcinoma

In HCC development and progression, the buildup of lactate and its increased concentrations are key factors contributing to the immunosuppressive characteristics of the TME (56). Lactylation, an emerging form of epigenetic modification, not only affects histones and transcription processes but is also widely present in non-histone proteins. Through proteomic analysis, Yang et al. identified 9,275 lactylation modification sites in HCC tissues, with 9,256 of these sites located in non-histone proteins, indicating that lactylation is a prevalent and extensive modification mechanism (57). In another study, Hong et al. identified 2,045 lactylation sites on 960 proteins and conducted a quantitative analysis of 1,438 sites across 772 proteins (58). These differentially lactylated proteins are involved in multiple key biological processes associated with HCC, including glycolysis, the tricarboxylic acid cycle, mitochondrial function, and cytoskeletal rearrangement, suggesting that lactylation is an important mechanism regulating metabolic reprogramming in HCC.

The elevation of lactylation levels not only promotes metabolic adaptation in tumor cells but also significantly enhances the immunosuppressive effect of the tumor microenvironment. For example, the study by Lu et al. demonstrated that lactate enhances the interaction with the TGF-β signaling pathway by regulating the lactylation of the Moesin protein in regulatory T cells (Tregs), thereby further promoting tumor immune escape (59). Furthermore, lactate and lactylation contribute to the immunosuppressive environment of HCC by altering immune cell functions and influencing the tumor microenvironment’s adaptability. This opens up new avenues for improving current immunotherapy approaches. For example, strategies targeting lactylation, such as inhibiting lactate production or interfering with lactylation-related signaling pathways, could significantly improve the effectiveness of immunotherapy and decrease the likelihood of tumor drug resistance.

Lactic acid and the lactylation modifications it induces are key regulatory elements in the metabolic reprogramming and immune suppression associated with HCC. Lactylation contributes to the initiation and progression of HCC by influencing essential biological processes, modulating immune cell activity, and adapting to the tumor microenvironment. Moving forward, targeting lactylation could offer promising approaches for enhancing immunotherapy in HCC.

### 3.5 Pancreatic cancer

**Figure 4:**
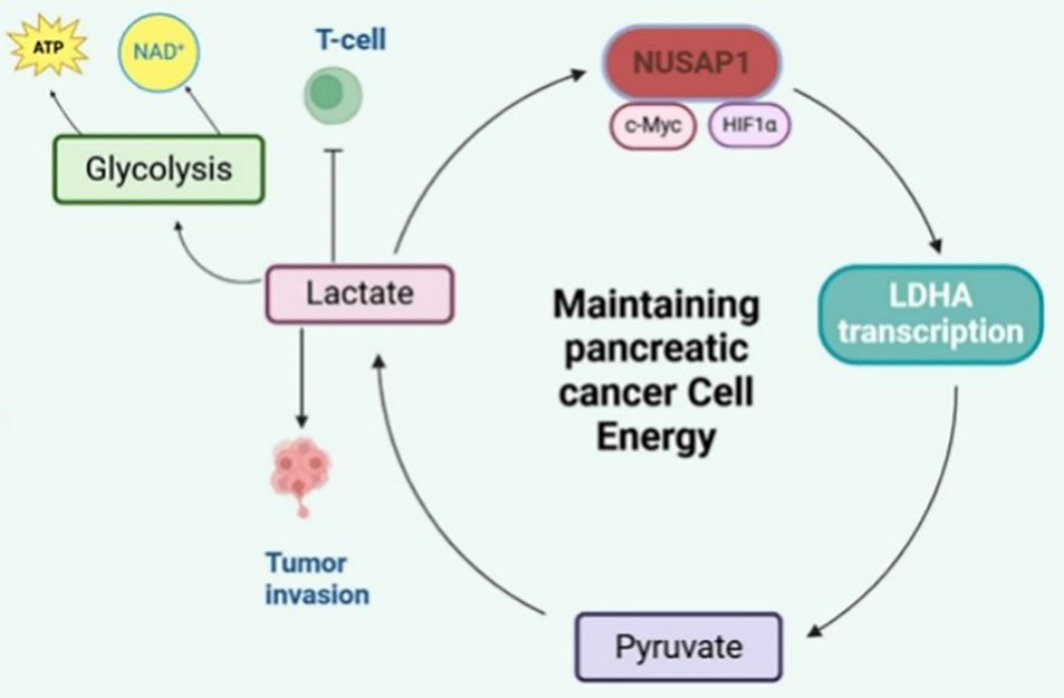
NUSAP1 and LDHA form a positive feedback loop in pancreatic cancer.

In PC, a significant signaling and epigenetic regulator is lactate, with lactylation being among the primary mechanisms involved (60). Particularly noteworthy is the formation, within PC’s glycolytic pathway, of a critical positive feedback loop by NUSAP1 and LDHA overexpression (61). A microtubule-associated protein, NUSAP1 interacts with transcriptional regulators c-Myc and HIF-1 (62). These factors, in turn, enhance LDHA promoter activity, leading to elevated LDHA expression levels. Essential for glycolysis promotion and lactate generation is LDHA, functioning as a core glycolytic enzyme. Stabilization of NUSAP1 occurs through lactylation-mediated inhibition of its degradation by lactate (61). Reinforced thereby is this feedback loop, sustaining continued glycolysis alongside lactate synthesis (60). Important molecular insights into the Warburg effect are thus provided by this mechanism (**Figure 4**). In addition, lactate accumulation within the pancreatic tumor microenvironment exerts immunosuppressive effects on CD8^+^ cytotoxic T lymphocytes (63). By entering T cells via MCT1, lactate causes intracellular acidification and disrupts the NAD^+^/NADH balance, thereby suppressing glycolytic activity and effector cytokine production such as IFN-γ (64, 65). Meanwhile, lactate reinforces tumor glycolysis through HIF-1α- and c-Myc-dependent upregulation of LDHA and MCT4, establishing a positive feedback loop that sustains energy production in PC cells and contributes to immune evasion (66).

In PC, NUSAP1 synergizes with c-Myc/HIF-1 to upregulate the expression of the key glycolytic enzyme LDHA, promoting lactate production. Lactate, in turn, stabilizes the NUSAP1 protein through lactylation modification, forming a positive feedback loop that drives sustained activation of glycolysis, elucidating the molecular mechanism of the Warburg effect.

Not merely as an energy substrate for PC cells’ rapid proliferation does lactate function, but also as a signaling molecule exerting influence upon tumor growth dynamics. Evidence has accumulated demonstrating that histone lactylation modifications, mediated by lactate, can induce chromatin structural alterations - genes relevant to pancreatic carcinogenesis thereby undergoing expression regulation. Particularly within microenvironments exhibiting elevated lactate concentrations, the chromatin remodeling processes facilitated by lactylation tend to upregulate oncogenic pathways activation (67, 68). Between metabolic reprogramming events and epigenetic modifications, this establishes lactate’s pivotal bridging role (69, 70). Through integrative analysis combining RNA-seq datasets with clinical outcomes, ten lactylation-associated genes (LRGs) of prognostic significance have been identified in pancreatic adenocarcinoma cases. Among these, transport-related SLC16A1 and immunomodulatory HLA-DRB1, along with potassium channel KCNN4, mitotic regulator KIF23 and metabolic enzyme HPDL, show survival correlation patterns with overall patient outcomes. Of particular note is SLC16A1’s central involvement - its lactate shuttling capacity directly modulates intracellular lactylation intensities. Experimental models both cellular and animal-based confirm that suppression of SLC16A1-mediated lactylation events markedly attenuates malignant progression parameters (71). Therapeutic potential therefore appears considerable regarding pharmacological targeting of this SLC16A1/lactylation axis in pancreatic oncology contexts.

Concluded it can be that lactate holds key importance-ways in PC cells’ metabolic demands fulfilling, with proteins such as NUSAP1 getting stabilized via lactylation-processes, thereby the Warburg effect maintaining. Evident from examples, tumor-related genes’ expression also gets influenced by lactate through epigenetic modifications-types. Innovative therapeutic approaches might emerge if the focus shifts to targeting lactate-dependent metabolic pathways and signaling mechanisms, with examples including LDHA inhibition or modulation of NUSAP1 and SLC16A1. Crucial for targeted treatments developing would be deeper explorations into how exactly lactate metabolism and its linked pathways contribute to PC progression, thus clinical outcomes potentially improving for patients in the end.

### 3.6 Gallbladder cancer

GBC is an uncommon yet highly aggressive cancer, with a five-year survival rate ranging from 5% to 10% (72). Although no studies have explicitly indicated a direct correlation between GBC and lactylation, existing research has demonstrated that elevated expression levels of glycolysis-related proteins are significantly associated with the progression of GBC (HR: 2.16, 95% CI: 1.70–2.75) (73). Glycolytic reprogramming is considered crucial in the initiation and development of GBC. In GBC tissues and cell lines, the expression of key glycolytic enzymes, such as glucose transporter GLUT1, lactate dehydrogenase LDHA, and pyruvate kinase pyruvate kinase M2 (PKM2), is markedly increased, accompanied by a significant rise in lactate production. This indicates that the energy metabolism of GBC cells heavily relies on the glycolytic pathway (74). Moreover, the elevated levels of LDH and lactate are not only closely associated with tumor progression in GBC but also significantly impact the poor prognosis of patients (75, 76). This metabolic feature could offer continuous energy support to GBC, while also boosting the tumor’s ability to invade.

From the viewpoint of molecular mechanisms, the pivotal glycolytic enzyme LDHA serves as a downstream target of miR-30d-5p. Research indicates that a reduction in miR-30d-5p levels mitigates its inhibitory effect on LDHA, thus improving the glycolytic capacity of GBC cells and facilitating their proliferation. In a similar manner, decreased expression of miR-181b-5p hampers the viability, migration, and glycolytic activity of GBC in hypoxic environments by elevating the levels of pyruvate dehydrogenase complex component X (PDHX) (77). Furthermore, miRNA-139-5p inhibits the processes of proliferation, migration, and glycolysis in GBC by targeting PKM2 (74). These results highlight the crucial role of miRNAs in regulating glycolysis in GBC, providing fresh insights for the development of future therapies targeting glycolysis.

Worth noting is that the rise in lactate metabolism-ways often appears alongside lactylation’s emergence, this newly recognized epigenetic modification-type being connected to multiple aggressive cancers. Key proteins’ modification by lactylation can influence metabolic reprogramming-processes and immune suppression-effects, thus it can be seen that GBC’s related mechanisms demand deeper investigation-work. Clinical retrospective studies-large in number have further shown serum and bile lactate levels-high, combined with tumor tissues’ LDHA expression-elevated, exhibit strong correlations with pathological grading-systems, clinical staging-criteria, and prognosis-outcomes in GBC cases (76, 78). Examples demonstrate these lactate metabolism-markers may serve as useful indicators for assessing disease severity-states and forecasting treatment results-potential in such malignancies.

The progression and development of GBC, closely associated with glycolytic reprogramming it is, where crucial roles in tumor invasion and prognosis are played by key enzymes of glycolysis along with lactate metabolism markers-ies (79). Unclear remains the direct lactylation-GBC relationship, yet existing evidence-ings suggest that significant impacts on tumor initiation and progression might be exerted by lactylation through modulation of metabolic reprogramming plus immune-suppressive mechanisms-es. Targeting lactate metabolism and lactylation, future research could contribute to a deeper understanding of GBC’s metabolic mechanisms, thereby providing new strategies for targeted therapy. For diagnosis, staging, and prognosis assessment, crucial evidence may be offered by lactate-related indicators-es, examples being the advancement of more precise clinical interventions in developments. To provide an at-a-glance comparison of lactylation-related targets, mechanisms and functional implications across digestive system tumors, we summarized current evidence in **Table 1**.

**Table 1:** Lactylation-related targets, mechanisms and functional implications in digestive system cancers.

Cancer type	Lactylation target(s)	Proposed mechanism	Impact on cancer	References
EC	SHMT2, Axin1, H3K9 (e.g., at LAMC2 promoter)	Hypoxia-induced lactate accumulation promotes lactylation of SHMT2 and Axin1, enhancing glycolysis and inhibiting DNA repair. Histone lactylation (e.g., H3K9la) activates oncogenic gene expression.	Promotes proliferation, migration, stemness, and chemotherapy resistance.	(33, 34)
GC	NSUN2, VCAM1, PD-L1, H3K18la	Lactylation regulates spliceosome function, ferroptosis resistance, immune checkpoint expression (PD-L1), and activates the AKT-mTOR signaling pathway.	Enhances cancer stemness, immune evasion, metastasis, and chemotherapy resistance.	(80, 81)
CRC	β-catenin, H3K18la, RUBCNL	Lactylation activates the Wnt/β-catenin signaling pathway, enhances autophagy, and promotes stemness. Histone lactylation (H3K18la) drives oncogenic transcription.	Drives tumor proliferation, metastasis, and resistance to therapies like bevacizumab.	(51)
HCC	PYCR1, ABCF1, Nucleolin, Moesin (in Tregs)	Lactylation reprograms metabolism, regulates RNA splicing, modulates TGF-β signaling, and alters immune cell function in the tumor microenvironment.	Promotes tumor growth, metastasis, immunosuppression, and drug resistance.	(59, 82–84)
PC	NUSAP1, LDHA, PKM2, Snail1, H4K12la	Forms a NUSAP1-LDHA positive feedback loop, enhancing glycolysis and epithelial-mesenchymal transition (EMT). Histone lactylation (H4K12la) supports metabolic adaptation.	Facilitates tumor proliferation, invasion, immune evasion, and metabolic adaptability.	(61, 66, 74, 85, 86)
GBC	LDHA, PKM2, PP4R1 (potential indirect target)	Upregulation of glycolytic enzymes (e.g., LDHA, PKM2) increases lactate production, potentially influencing lactylation-mediated metabolic and signaling pathways.	Promotes tumor growth, invasion, and chemotherapy resistance.	(52, 74, 78)

## 4 The clinical significance of lactylation in digestive system tumors

Dynamic changes in lactylation levels can serve as crucial biomarkers for early disease diagnosis and monitoring disease progression (52, 87). Elevated lactylation, particularly histone H3K18 lactylation, is associated with poorer prognosis in digestive system tumors like GC. The distinct patterns of lactylation across different tumors suggest its potential as a biomarker for predicting patient responses to chemotherapy and immunotherapy (88).

Particularly in oncology, leveraging bioinformatics tools and big data analytics to integrate lactylation status information holds promise for constructing more efficient and precise diagnostic and detection models, thereby providing significant support for disease prevention and treatment (89, 90). Targeting lactylation has been identified as a potential novel strategy for treating digestive system tumors (91). Lactylation is considered an emerging and promising target in digestive system tumor research and therapy. Targeting lactylation provides innovative strategies to overcome drug resistance and improve cancer treatment outcomes (92–94). Lactylation plays a pivotal role in regulating tumor metabolism, immune evasion, and drug resistance, underscoring the importance of developing potent lactylation inhibitors (95). These inhibitors can modulate lactylation levels and intervene in the key metabolic and signaling pathways of tumor cells, thereby inhibiting tumor growth and metastasis. On this basis, applying lactylation inhibitors in clinical trials to verify their safety and therapeutic efficacy not only opens new possibilities for treating digestive system tumors but may also promote the further development of precision medicine in this field.

### 4.1 Esophageal cancer

#### 4.1.1 Diagnostic and prognostic indicators

The research conducted by Shi et al. demonstrated that the long non-coding RNA AP001885.4 plays a significant role in promoting both the proliferation and invasion of esophageal squamous cell carcinoma (ESCC) cells through the regulation of histone lactylation, transcriptional activation dependent on NF-κB (p65), and the stability of c-myc mRNA mediated by METTL3 (41). The elevated levels of AP001885.4 emphasize its essential involvement in tumor progression and highlight its potential as both a diagnostic marker and a therapeutic target. Zhang et al. observed a significant increase in lactate levels in ESCC cells under hypoxic conditions, leading to a marked rise in histone lysine lactylation (Kla), particularly at the H3K9 site. The lactylation at H3K9 significantly enhances the proliferative and invasive capabilities of ESCC cells by upregulating the expression of the LAMC2 gene, which is involved in cell adhesion and migration (33). These findings highlight the importance of hypoxia-induced lactylation modifications in cancer and support the potential of LAMC2 as a diagnostic biomarker. Additionally, Yao et al. showed that lactylation of the serine hydroxymethyltransferase 2 (SHMT2) protein under low oxygen conditions not only stabilized the protein but also promoted the proliferation, migration, invasion, stemness, and glycolytic metabolism of ESCC cells (34). The high expression of SHMT2 in EC tissues and its critical role in tumor progression make it a promising candidate for use as a diagnostic marker.

#### 4.1.2 Therapeutic targets

Studies have shown that in hypoxic environments, the buildup of lactate leads to the lactylation of the Axin1 protein, especially at lysine 147 (K147). This lactylation modification diminishes the functionality of Axin1 by facilitating its ubiquitination and subsequent degradation (39). The degradation of Axin1 further activates glycolysis-related signaling pathways, thereby enhancing the glycolytic capacity of EC cells and promoting the survival and invasion of tumor cells at the metabolic level. This finding suggests that targeted interventions against Axin1 lactylation may offer new therapeutic targets and strategies for EC treatment through the regulation of tumor metabolism.

### 4.2 Gastric cancer

#### 4.2.1 Diagnostic and prognostic indicators

In the study of gastric adenocarcinoma cells, a total of 1,014 lactylated proteins and 2,375 lactylation sites were identified, which were significantly enriched in pathways related to spliceosome function. Compared to adjacent tissues, the level of lactylation was higher in GC tissues and was closely associated with poor prognosis (43, 46). The lactylation score model constructed based on lactylation-related genes demonstrated a significant correlation with overall survival (OS) and disease progression in GC patients; those with high lactylation scores exhibited a worse prognosis and a diminished response to immune checkpoint inhibitor (ICI) therapy, suggesting that lactylation may influence the efficacy of immunotherapy (96). In GC cells, SIRT1 downregulates the expression of H19 through delactylation and modulates the lactylation level of histone H3K18, thereby affecting gene transcription and cellular metabolism (97). SIRT1 also reprograms the metabolic state of GC cells by regulating glycolysis and lactate metabolism, further promoting tumor progression. This suggests that H19, SIRT1, and the H3K18 lactylation modifications they regulate may serve as valuable diagnostic markers, offering insights into patients’ metabolic profiles and disease progression. Additionally, METTL14 reduces ATF5 expression through m6A modification, which inhibits the WDR74/β-catenin axis and diminishes the stemness properties of GC cells (98). The expression level of METTL14 is closely associated with tumor stemness and progression, indicating its potential as a critical marker for monitoring GC development. MFAP5-positive cancer-associated fibroblasts (CAFs) promote the invasiveness and metastatic potential of GC cells by facilitating epithelial-mesenchymal transition (EMT), with elevated MFAP5 expression potentially serving as an important marker for GC staging and diagnosis (99, 100). Furthermore, lactylation of NSUN2 enhances its activity by regulating GCLC-dependent glutathione synthesis, which supports cancer cell resistance to ferroptosis (69, 101). The expression and lactylation status of NSUN2 reflect the metabolic characteristics and ferroptosis resistance of tumors, offering substantial diagnostic and prognostic value.

#### 4.2.2 Therapeutic targets

**Figure 5:**
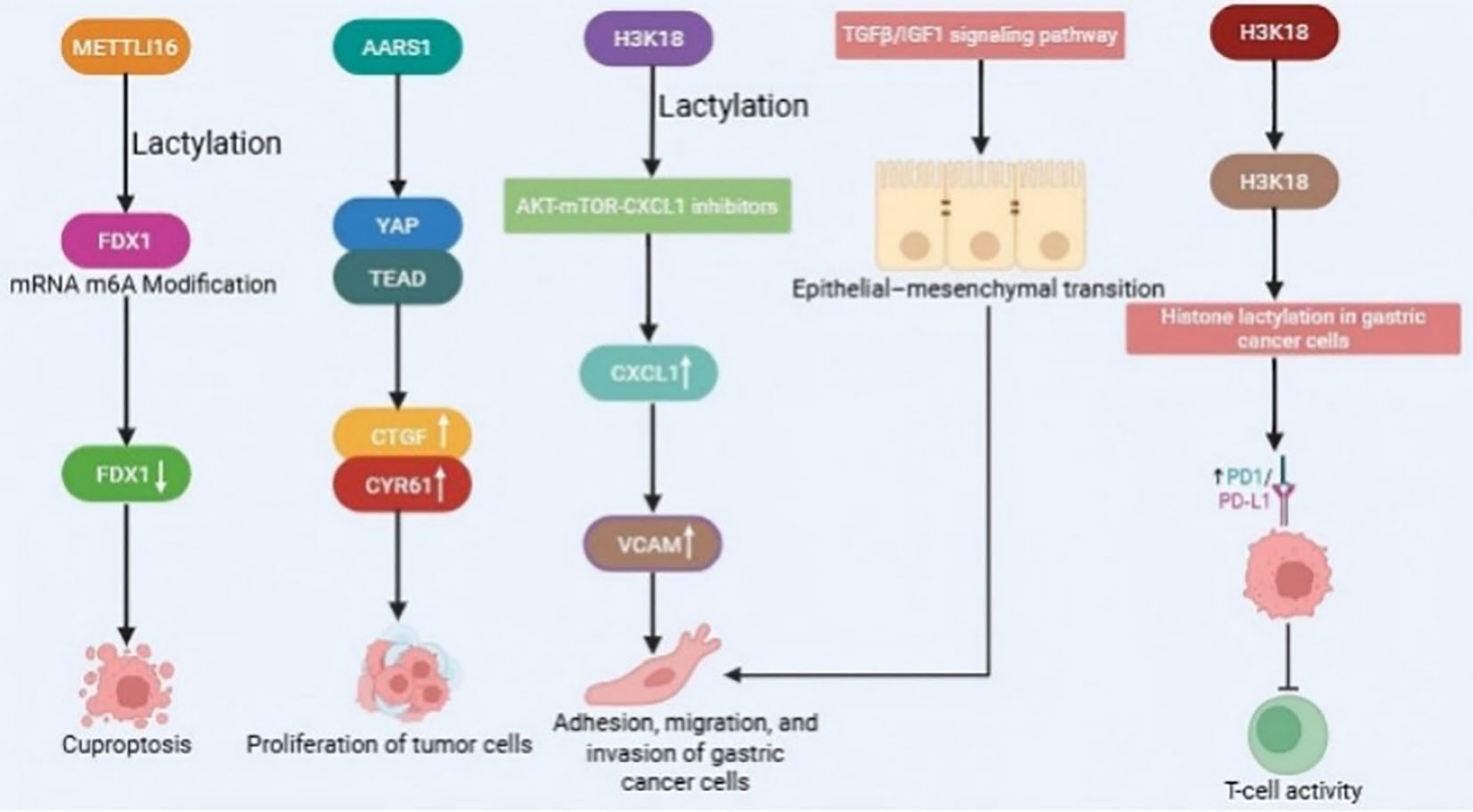
The multifaceted oncogenic mechanisms of lactylation in gastric cancer.

Research indicates that lactylation modification plays a critical role in the initiation and progression of GC, presenting a novel target for therapeutic intervention. METTL16 regulates lactylation levels in GC cells by modifying FDX1 mRNA via m6A, affecting the stability of FDX1 mRNA and activating the cuproptosis mechanism (101). This suggests that METTL16 could be a potential therapeutic target for GC. Additionally, AARS1, a key enzyme involved in lactylation, is crucial for activating the YAP signaling pathway. This highlights AARS1’s role not only in amino acid synthesis but also in metabolic regulation in GC (102). The modulation of YAP activity through lactylation may provide an innovative approach to GC treatment. Moreover, H3K18 lactylation has been shown to promote GC progression and metastasis by increasing VCAM1 expression. This modification regulates VCAM1 through the AKT-mTOR-CXCL1 signaling pathway, enhancing the adhesion, migration, and invasion of GC cells, thus providing direct evidence of lactylation’s role in GC metastasis (80). Moreover, LOX released by CAFs enhances PD-L1 expression through histone lactylation, which aids GC cells in escaping immune surveillance and regulates the EMT of these cells via the TGFβ/IGF1 signaling pathway, further augmenting the invasive and metastatic capacity of tumors (81). Collectively, these investigations unveil the diverse roles of lactylation modification in the metabolic regulation, immune evasion, and invasive metastasis of GC, highlighting potential targets and innovative strategies for its treatment (**Figure 5**).

This figure illustrates the central role of lactylation modification in metabolic reprogramming, immune evasion, and metastasis of GC. ① METTL16 regulates the stability of FDX1 mRNA through m6A modification, affecting the level of lactylation and cuproptosis. ② AARS1, as a key enzyme for lactylation, activates the YAP signaling pathway. ③ Histone H3K18 lactylation upregulates VCAM1 through the AKT-mTOR-CXCL1 axis, enhancing cancer cell migration and invasion. ④ LOX released by CAFs upregulates PD-L1 expression by inducing histone lactylation to aid tumor immune evasion and regulates EMT through the TGFβ/IGF1 pathway, collectively enhancing the tumor’s invasive and metastatic capabilities.

#### 4.2.3 Therapeutic drugs

Recent studies have demonstrated that Stiripentol can effectively inhibit lactic acid production in GC cells and block lactylation modifications at the K388 site of NBS1. This inhibition reduces DNA repair efficiency and helps overcome tumor resistance to both radiotherapy and chemotherapy. These findings have been validated using patient-derived organoid models and xenograft models. Research indicates that when Stiripentol is used in combination with cisplatin or ionizing radiation, it exhibits significant synergistic effects. This combination not only diminishes the DNA repair capacity of tumor cells but also significantly enhances the therapeutic efficacy of radiotherapy and chemotherapy. Consequently, this combination therapy effectively suppresses tumor growth and extends the survival of mice in experimental settings, indicating strong potential for future clinical applications (103).

### 4.3 Colorectal cancer

#### 4.3.1 Diagnostic and prognostic indicators

In the field of CRC research, crucial remains the identification of both effective treatment approaches and reliable markers for diagnosis and prognosis. Examined by Domb et al. were the impacts of CXCR4 siRNA/dextran-spermine nanoparticles on CXCR4 expression and serum LDH levels, using a mouse model with liver metastasis from CRC. Suggested by their findings is that targeting CXCR4 might alter the metastatic tendencies of cancer cells, thus it can be seen a potential therapeutic strategy for controlling metastasis in CRC. Also observed was an association between CXCR4 suppression and decreased serum LDH levels, examples indicating LDH could serve as a useful biomarker for tracking disease progression, offering significant implications for clinical evaluation purposes (104).

Recent years have witnessed growing interest among researchers regarding lactylation modifications’ role in CRC-ology. Frequently detected are elevated lactylation levels within tumor tissues or circulating blood samples from affected patients, these biochemical alterations showing clear associations with disease burden. Illustrative of this phenomenon is H3K18la, whose mass spectrometry-based detection methodology might offer diagnostic possibilities non-invasively. When integrated with conventional biomarkers, such as CEA and CA19-9, diagnostic sensitivity can be enhanced, particularly for early-stage lesion identification or monitoring minimal residual disease. Correlated strongly with adverse clinical outcomes are high lactylation concentrations, as demonstrated by their frequent coexistence with aggressive pathological characteristics: lymph node metastasis occurring alongside vascular invasion events, such manifestations typically heralding unfavorable patient prognoses (51).

Exploring the underlying mechanisms, lactylation modification may contribute to tumor progression by activating oncogenic signaling pathways, such as HIF-1α and MYC. Additionally, it could worsen the immunosuppressive tumor microenvironment by impairing anti-tumor immune responses, for example, by altering T cell function. Studies have shown that lactylation modification in the tumor microenvironment not only affects the metabolism of tumor cells but may also promote tumor growth and metastasis by regulating the function of immune cells (105).

Lactylation modification holds significant potential application value in the diagnosis and prognosis assessment of CRC, opening new avenues for research and clinical practice in this field.

#### 4.3.2 Therapeutic targets

Recent discoveries in CRC studies continue to uncover promising therapeutic targets, sparking fresh optimism against this formidable malignancy. Of particular interest is Proprotein Convertase Subtilisin/Kexin Type 9(PCSK9), identified by Yu et al. as playing a critical role in colon cancer development. This protein is responsible for tumor progression and metastasis through its involvement in essential biological mechanisms like EMT, a process that enables cancer cells to migrate and invade surrounding tissues. The PI3K/AKT signaling cascade, which is central to controlling cell survival, proliferation, and movement, is also affected by PCSK9. Studies show that this interaction significantly enhances the proliferative and metastatic capabilities of malignant cells. Further complicating the tumor microenvironment is PCSK9’s ability to alter macrophage polarization, thereby creating conditions favorable for tumor growth. Thus, targeting PCSK9 presents substantial therapeutic potential; it may curtail metastasis, delay disease progression, and modulate immune responses within the tumor microenvironment. A promising avenue for future investigation is the development of PCSK9 inhibitors as an innovative strategy for treating colon cancer (106).

The discovery by Jiang et al. is significant, as it goes beyond PCSK9 to reveal the NSUN2/YBX1/m5C-ENO1 feedback loop as a potential therapeutic target in CRC treatment. This regulatory mechanism, which is crucial for CRC progression, operates through alterations in metabolic pathways. The methyltransferase NSUN2, by mediating m5C modifications, enhances the stability of ENO1, a glycolytic enzyme crucial for energy production. YBX1 is involved in this stabilization process, forming a self-reinforcing cycle that amplifies glycolytic activity (107). Studies show that this metabolic reprogramming directly supports the proliferative demands characteristic of malignant cells. This feedback loop not only satisfies the energetic needs of tumors but also enhances their metastatic potential.

The study conducted by Ma and colleagues primarily investigates immunotherapy approaches for cancer, with PD-L1 lactylation being identified as a crucial therapeutic target. Their research demonstrates the dual role of this mechanism; it not only strengthens the body’s immune response against tumors but also promotes the evasion of immune surveillance. Observed in the findings was that diets lacking serine and glycine influence tumor development. Enhanced is the activity of anti-tumor immunity under such dietary conditions, while simultaneously enabled is immune escape through the lactylation of PD-L1. Critical in immune regulation, this protein serves as a checkpoint. PD-L1 lactylation stabilizes its expression on the surface of tumor cells, enabling tumors to evade immune surveillance by inhibiting T cell-mediated responses. Targeting this process presents a promising therapeutic strategy, as inhibiting PD-L1 lactylation can reduce its expression and restore T cell activity, thereby enhancing the efficacy of immune checkpoint blockade therapy (108). Furthermore, combining metabolic interventions (such as a serine/glycine-free diet) with immune checkpoint inhibitors may further improve clinical outcomes.

Additional studies have revealed that METTL1 regulates PKM2 expression through m7G modification, subsequently activating CD155 and promoting immune evasion in CRC, thus providing a new potential target for prognosis evaluation and immunotherapy in CRC (109). These continuously emerging research findings elucidate the pathogenesis of CRC and potential therapeutic targets from various perspectives, offering an important theoretical basis for the future development of immunotherapy and cancer treatment strategies.

#### 4.3.3 Therapeutic drugs

Lactylation serves various functions in the development, advancement, and resistance to treatment of CRC. This modification facilitates malignant transformation and the emergence of drug resistance in CRC by influencing tumor cell metabolism, epigenetic alterations, and mechanisms of immune evasion. Strategies that aim to target lactylation may open up new avenues for CRC therapy by obstructing lactylation modifications and disrupting signaling pathways driven by lactate. For example, research has shown that inhibiting crucial enzymes associated with lactylation, such as LDHA, can decrease lactate buildup, hinder the metabolic reprogramming of cancer cells, and consequently improve the effectiveness of chemotherapy and targeted therapies (51). Specifically, by addressing inhibitors of lactylation modifications, it is possible to restore normal cellular metabolism, thus reducing tumor cell resistance to anticancer medications like Bevacizumab and significantly enhancing the therapeutic efficacy of these treatments. To facilitate a clearer understanding, therapeutic agents that modulate lactylation and their mechanisms of action are summarized in **Table 2**.

**Table 2:** Therapeutic agents targeting lactylation in cancer treatment.

Drug name	Target/Mechanism of action	Associated cancer types	Potential effects/Authors’ insights	Development stage	References
Stiripentol	Inhibits lactate production and specifically blocks lactylation modification at the K388 site of the NBS1 protein.	GC	Impairs DNA damage repair and, when combined with cisplatin or radiotherapy, markedly reverses chemo- and radio-resistance. Authors’ insight: STP represents a novel therapeutic approach that bridges metabolic regulation (lactate metabolism) and genomic stability (DNA repair).	Preclinical study (patient-derived organoids and mouse models)	(103)
Oxamate	Directly inhibits the activity of LDHA.	PC, CRC, and others	Reduces lactate production, inhibits glycolysis, and reverses the Warburg effect, thereby decreasing lactylation levels. Authors’ insight: As a classic LDHA inhibitor, it serves as a valuable tool compound for studying lactylation, though its druggability remains to be improved.	Preclinical study	(51, 110)
Demethylzeylasteral	Targets lactate metabolism and histone lactylation.	Liver cancer	Inhibits the tumorigenic addiction of liver cancer stem cells. Authors’ insight: This agent demonstrates that simultaneously targeting lactate metabolism and its downstream epigenetic effects represents a promising strategy to overcome tumor stemness and therapeutic resistance.	Preclinical study	(111)
LDHA inhibitors (various agents under development)	Inhibits LDHA, thereby reducing lactate production at its source.	Broadly applicable (e.g., CRC, liver cancer, etc.)	Reduces tumor microenvironment acidity and intracellular lactylation levels, suppressing tumor growth and enhancing sensitivity to chemotherapy and targeted therapy. Authors’ insight: Represents an upstream strategy for targeting the lactylation pathway; however, potential metabolic side effects on normal tissues should be carefully considered.	Preclinical and early clinical development	(51, 112)
MCT inhibitors (e.g., AZD3965)	Inhibits MCTs and blocks lactate efflux.	Broadly applicable (e.g., PC)	Causes intracellular lactate accumulation, leading to feedback inhibition of glycolysis and alteration of the tumor microenvironment. Authors’ insight: Complementary to LDHA inhibitors, it represents another key regulatory node in the lactylation pathway and may be particularly effective in tumors with high SLC16A1 expression.	Partially entered clinical trials	(32, 71)
SCH772984	Inhibits the ERK1/2 signaling pathway.	GBC	When combined with PP4R1 inhibition, it exhibits synergistic inhibitory effects against GBC. Authors’ insight: This case suggests that integrating conventional signaling pathway inhibitors with strategies targeting lactylation-related proteins (such as PP4R1) may achieve improved therapeutic efficacy.	Preclinical study	(52)

### 4.4 Hepatocellular carcinoma

#### 4.4.1 Diagnostic and prognostic indicators

**Figure 6:**
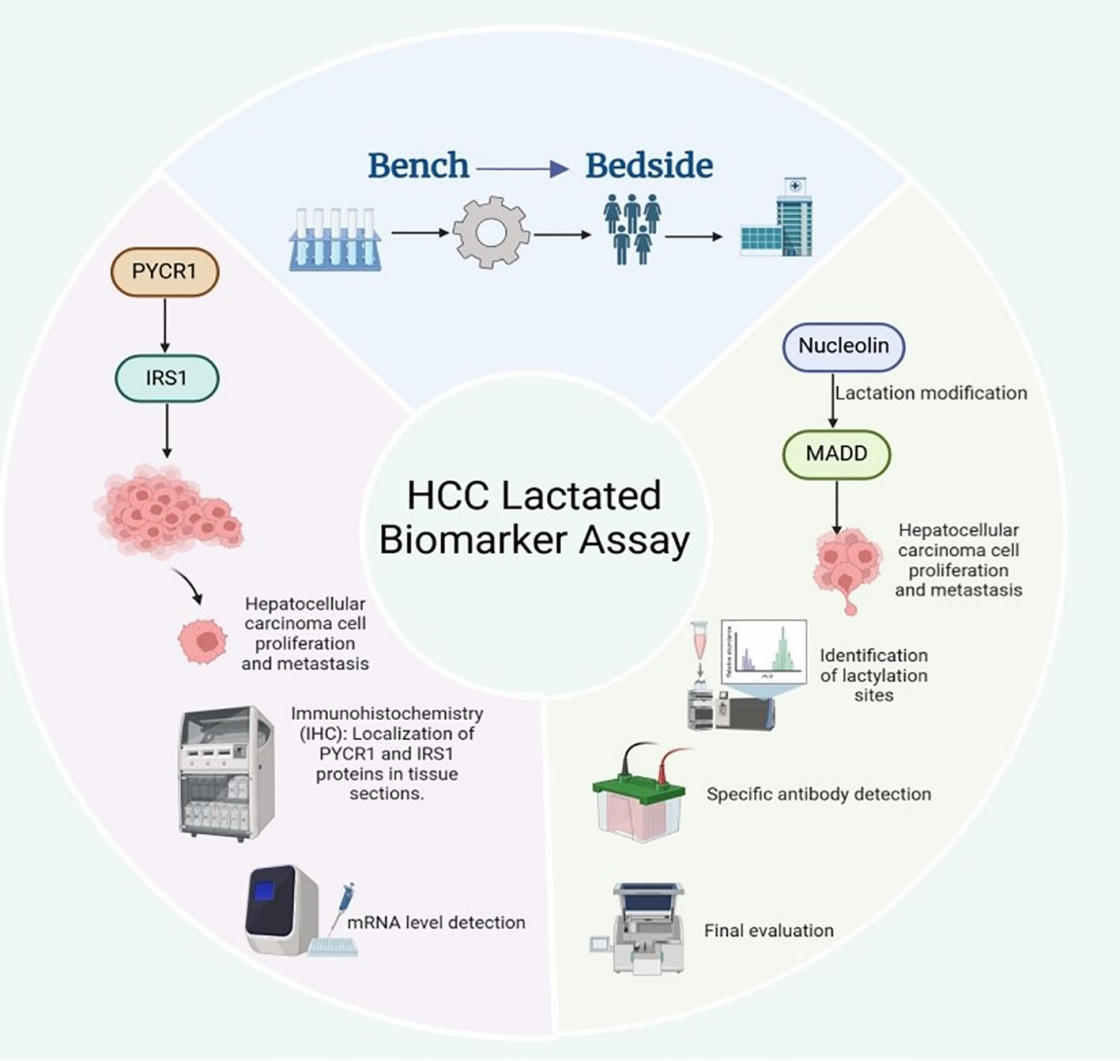
Potential diagnostic and prognostic biomarkers for liver cancer.

Li et al. indicates that the expression levels of PYCR1 and IRS1 may serve as potential diagnostic biomarkers for liver cancer (82). PYCR1 promotes the growth and metastasis of liver cancer cells by regulating IRS1 expression. Thus, measuring the levels of these two molecules can aid in assessing the presence and progression of liver cancer. Currently, the detection of PYCR1 and IRS1 levels primarily relies on immunohistochemistry (IHC) and quantitative PCR (qPCR) techniques. Some small-scale clinical studies have validated their potential value in the early diagnosis of liver cancer. However, the clinical application of these markers requires further large-scale, multicenter clinical trials to determine their specificity and sensitivity across different populations. In addition, Tao et al. found that the lactylation modification of Nucleolin may serve as an early diagnostic biomarker for intrahepatic cholangiocarcinoma, and this modification can regulate the expression of MADD (83). Currently, Nucleolin lactylation can be detected through liquid chromatography-tandem mass spectrometry (LC-MS/MS) or Western blot analysis using specific antibodies to determine its modification patterns in intrahepatic cholangiocarcinoma. Meanwhile, MADD expression levels can be assessed using qPCR or ELISA, and its specific splice variants are expected to serve as prognostic indicators for intrahepatic cholangiocarcinoma, aiding in evaluating tumor malignancy and metastatic potential (83). However, these methods remain in the laboratory research phase, and standardized clinical testing protocols and large-scale validation studies are yet to be established (**Figure 6**).

Prospects of Lactylation and Related Molecules as Biomarkers for HCC. In HCC, PYCR1 drives tumor growth and metastasis by upregulating IRS1; the protein and mRNA levels of PYCR1 and IRS1 (detected by IHC and qPCR, respectively) can serve as a potential diagnostic combination, but their clinical application still requires large-scale trials to verify their specificity and sensitivity. In intrahepatic cholangiocarcinoma, lactylation modification of nucleolar proteins (detected by LC-MS/MS or specific antibody Western blot) is a promising early diagnostic marker; this modification regulates the expression of the downstream MADD gene, and specific splice variants of MADD (assessed by qPCR or ELISA) are expected to be used for evaluating tumor malignancy and metastatic potential, serving as independent prognostic indicators.

Additionally, given Nucleolin’s regulatory role in RNA splicing, specific RNA splicing variants may play a crucial role in intrahepatic cholangiocarcinoma. RNA sequencing (RNA-seq) has been utilized to analyze splicing variants in intrahepatic cholangiocarcinoma samples, and these splicing signatures could potentially serve as novel biomarkers for predicting tumor progression and treatment response. However, the high costs and complex data analysis requirements of RNA-seq have limited its clinical adoption. Future efforts should focus on developing more straightforward and cost-effective detection methods, such as digital PCR (dPCR) or specific RNA probe assays, to facilitate clinical translation. Previous studies have shown that HDAC1 and HDAC2 are frequently overexpressed in hepatocellular carcinoma and that higher expression levels are associated with poorer survival, suggesting their potential value as prognostic biomarkers and therapeutic targets in HCC (113, 114). Currently, the detection of HDAC1 and HDAC2 primarily relies on IHC and Western blot techniques, which have been validated in some HCC patient samples. However, there remains a lack of standardized detection protocols for HDAC1/2 in clinical settings, and inhibitors targeting them as therapeutic options (such as HDAC inhibitors) are still in the early stages of clinical trials in HCC.

In summary, the detection of PYCR1, IRS1, Nucleolin lactylation, MADD, HDAC1, and HDAC2 holds potential clinical value in early diagnosis, prognosis assessment, and personalized treatment of liver cancer. Nevertheless, the clinical application of most of these markers remains in the research stage, necessitating further large-scale clinical studies to validate the feasibility of their detection methods, standardized protocols, and their application value across different populations.

#### 4.4.2 Therapeutic targets

In the context of overcoming lenvatinib resistance in HCC, Zhang et al. has demonstrated that Insulin-like Growth Factor 2 Binding Protein 3(IGF2BP3) plays a pivotal role in HCC’s resistance to lenvatinib, positioning it as a potential therapeutic target (115, 116). Inhibitors that target IGF2BP3 may aid in overcoming drug resistance, thereby enhancing the therapeutic efficacy of lenvatinib. Currently, preclinical studies have shown that IGF2BP3 inhibitors possess potential in suppressing HCC growth and overcoming drug resistance in mouse models. However, larger-scale animal experiments and clinical trials are necessary to validate their feasibility and efficacy in clinical settings. Promising appears the therapeutic strategy targeting lactylation regulation alongside serine metabolism manipulation. Metabolic reprogramming processes that contribute to drug resistance development can be disrupted through such pathway interventions. Examples demonstrate the enhanced efficacy of lenvatinib, highlighting its potential. Preclinical trials have shown that lactylation inhibitors can obstruct resistance-linked metabolic mechanisms, although their practical applications still require extensive clinical validation.

Noteworthy appears the involvement of m6A modifications-ways in HCC drug resistance phenomena, wherefrom emerges the therapeutic potentiality of m6A-targeting agents for resistance reversal and lenvatinib efficacy enhancement. Demonstrated through cellular models plus animal experimentation-data, inhibitors modifying m6A show effectiveness against lenvatinib resistance scenarios, examples suggesting their viability as forthcoming treatment modalities for HCC resistance management. In addition, the currently identified therapeutic targets associated with lactylation modification in hepatocellular carcinoma are summarized in **Table 3**.

**Table 3:** Therapeutic targets of lactylation modification in hepatocellular carcinoma.

Researcher	Finding	Contents	Potential applications	Reference
Yang et al.	Glypican-3 (GPC 3) hinders the growth of HCC cells in the hypoxic microenvironment by inhibiting lactfication modification	Cell experiments showed that up-regulation of GPC 3 significantly inhibited HCC cell proliferation and migration	GPC 3 could be used as a potential therapeutic target for HCC	(117)
Yuan et al.	ABCF1 Promote HCC progression by modification of K430 lactlation	The ABCF1 inhibitor significantly retarded tumor growth and metastasis by inhibiting its lactfication modification in animal models	Targeting ABCF1 or its lactactic modification may become a novel strategy to inhibit HCC growth and metastasis	(84)
Li rt al.	PYCR1 Is highly expressed in HCC cells, regulates IRS 1 through lactate modification, and promotes HCC cell proliferation, migration, and metastasis	Preclinical studies have shown that inhibition of PYCR1 expression effectively reduces the proliferation and migration of HCC cells	PYCR1 May become a therapeutic target for HCC and provide a new perspective for clinical research	(82)
Liu et al.	CA3 has a function of inhibiting tumor cell growth, and degalactic modification weakens its anticancer effect	Dellactification modification was found to weaken the anticancer effect of CA3 by restoring the exc	The role of lactification in regulating CA3 function provides novel targets for HCC therapy	(118)

Recent studies have further confirmed that IGF2BP3, PCK2, NRF2, and m6A modification play significant roles in the mechanism of lenvatinib resistance in HCC, making them potential therapeutic targets for overcoming drug resistance. Preclinical studies have demonstrated that inhibitors of IGF2BP3 and PCK2 can effectively enhance the efficacy of lenvatinib, while regulatory drugs for m6A modification are under development with the aim of restoring drug sensitivity. These findings provide new directions for precision therapy in liver cancer resistance; however, more extensive clinical validation is still needed to assess their practical clinical application effects (115, 119).

#### 4.4.3 Therapeutic drugs

Currently, researchers are actively exploring targeted therapies that focus on lactylation. Demethylzeylasteral is considered a potential therapeutic agent that inhibits the tumorigenicity of liver cancer stem cells by targeting lactate metabolism and histone lactylation. Therefore, Demethylzeylasteral or similar compounds may become new treatment options for liver cancer. Current investigations have highlighted the pivotal role of lactate metabolism regulation and histone lactylation modifications in the metabolic reprogramming of liver cancer stem cells, creating new therapeutic targeting possibilities (111). Demethylzeylasteral is still at the preclinical stage, with clinical trial phases yet to begin. Previous research has demonstrated its capacity to effectively suppress the proliferative and migratory behaviors of liver cancer stem cells through modulation of lactate metabolism and reduction of histone lactylation in HCC. Preliminary findings are encouraging, but further verification is necessary regarding demethylzeylasteral’s safety profile and treatment efficacy. Animal model experiments indicate this compound’s relatively low toxicity, but extensive human clinical testing must precede its application to thoroughly assess safety parameters, pharmacokinetic properties, and therapeutic outcomes.

Besides demethylzeylasteral, which suppresses liver cancer stem cell tumorigenicity by inhibiting histone lactylation, other therapeutic approaches targeting lactylation are being actively explored, including agents that reduce lactate production via LDHA inhibition or interfere with lactate transport and lactyltransferase activity, highlighting lactylation-related pathways as promising anticancer strategies (120). Research has found that LDHA inhibitors exhibit significant anti-tumor effects in certain tumor types and are undergoing preclinical validation. Although these drugs are currently in the early stages, they demonstrate the potential to treat liver cancer and other malignant tumors by regulating metabolic pathways through lactylation modification.

### 4.5 Pancreatic cancer

#### 4.5.1 Diagnostic and prognostic indicators

Xu et al. developed a lactylation-related scoring model using gene expression data and bioinformatics approaches, and showed that the resulting signature was associated with overall and disease-free survival in patients with breast cancer. In pancreatic adenocarcinoma, a separate lactylation-related prognostic signature was constructed by combining the expression levels of five lactylation-related genes into a weighted risk score, and patients with higher scores had significantly poorer overall survival (71).

The clinical application of lactylation as a biomarker is still limited. Although high-throughput gene expression profiling, bioinformatics analyses, and immunohistochemistry can provide informative diagnostic data, these methods are technically demanding and costly. They often require high-quality tissue samples, complex analytical pipelines, and specialized antibodies and equipment, which restricts their feasibility in routine clinical practice.

In this context, Zheng et al. integrated multiple machine learning approaches, including LASSO regression, XGBoost, and random forest, with bulk and single-cell transcriptomic data to identify hypoxia- and lactylation-related genes in pancreatic ductal adenocarcinoma. On the basis of these genes, they defined two molecular subtypes with distinct survival profiles and constructed a risk score that showed predictive value for overall survival and for modeled responses to immunotherapy and chemotherapy in PDAC patients. This hypoxia–lactylation-related signature, together with the identification of CENPA as a potential therapeutic target, provides a useful framework for linking lactylation biology to prognosis and treatment stratification in PDAC (121).

Hou and colleagues examined H3K18la expression in pancreatic cancer using Western blot analysis of tumor and adjacent non-tumor tissues. They found that both global protein lactylation and H3K18la levels were higher in pancreatic cancer tissues than in para-carcinoma tissues. H3K18la expression in tissue showed positive correlations with serum lactate, CA19-9, and CEA (r = 0.774, 0.744, and 0.589, respectively). In addition, serum H3K18la yielded an area under the ROC curve of 0.848 for distinguishing pancreatic cancer, indicating promising diagnostic performance. These findings suggest that histone H3K18 lactylation may serve as a potential biomarker for the diagnosis and severity assessment of pancreatic cancer (85).

The diagnostic and prognostic value of lactylation markers in pancreatic cancer is encouraging, but their clinical use is not yet ready. Standardized detection protocols, wider access to the necessary technology, and lower costs are essential before implementation in routine practice. Future work should focus on simplifying and harmonizing assays so that they are affordable, reproducible, and scalable across laboratories. Ultimately, the utility of lactylation-based biomarkers will depend on overcoming these technical and logistical barriers.

#### 4.5.2 Therapeutic targets

Building on these findings, Wang et al. used a multi-omics strategy, integrating bulk and single-cell RNA sequencing, metabolomics, ATAC-seq and CUT&Tag, to dissect histone lactylation in pancreatic ductal adenocarcinoma. They identified an LDHA–H4K12la–immune-gene axis that links glycolytic metabolism, epigenetic regulation and immune signaling, highlighting this pathway as a potential target for therapeutic intervention in pancreatic cancer. Their data suggest that interfering with LDHA-driven lactate production or H4K12 lactylation could help disrupt pro-metastatic metabolic and immunologic circuits, underscoring the broader relevance of lactylation to PDAC progression (122).

Moreover, a recent study showed that overexpression of the small GTPase RHOF in pancreatic cancer cells upregulated c-Myc, which in turn enhanced PKM2 transcription, augmented glycolysis, and increased lactate production. The elevated lactate promoted lactylation of the transcription factor Snail1, facilitated its nuclear translocation, and ultimately drove epithelial–mesenchymal transition. These findings highlight RHOF, c-Myc, PKM2, and Snail1 as key components of a lactylation-related glycolytic axis in pancreatic cancer and suggest that this pathway may represent a potential target for therapeutic intervention, particularly in tumors with activated glycolysis (86).

In pancreatic ductal adenocarcinoma, Liu et al. showed that CTCF forms a complex with FLG-AS1 and HNRNPU to recruit EP300 to the IGF2BP2 promoter. This recruitment induces histone lactylation at the IGF2BP2 locus, increases IGF2BP2 expression, and promotes PDAC cell proliferation, pointing to the CTCF–EP300–IGF2BP2 axis as an important lactylation-related regulatory node and a potential therapeutic entry point (123).

Huang et al. reported that lactate in the tumor microenvironment drives EP300-dependent lactylation of NMNAT1 at lysine 128, enhancing its nuclear localization and enzymatic activity. This modification maintains the nuclear NAD salvage pathway and supports tumor cell survival under glucose deprivation, suggesting that NMNAT1 and its associated metabolic circuitry may also represent attractive targets within the lactylation network of pancreatic cancer (124).

#### 4.5.3 Therapeutic drugs

LDHA inhibitors such as oxamate and FX11 reduce lactate production and attenuate glycolytic flux in PDAC models, leading to broad changes in metabolic pathway activity and, in some settings, a shift toward oxidative metabolism. Integrative transcriptomic and metabolomic analyses of oxamate-treated and oxamate-resistant PDAC cells have shown coordinated alterations in glycolytic enzymes, the pentose phosphate pathway and fatty acid metabolism, illustrating how PDAC cells rewire their metabolic network to adapt to LDHA inhibition (110, 125).

Several preclinical studies have shown that pharmacological inhibition of LDHA can suppress glycolytic flux, reduce lactate production, and inhibit pancreatic cancer growth *in vitro* and *in vivo*, supporting LDHA and lactate metabolism as potential therapeutic targets rather than established clinical options (60).

### 4.6 Gallbladder cancer

#### 4.6.1 Diagnostic and prognostic indicators

At present, no studies have directly examined lactylation in gallbladder cancer (GBC). GBC is a highly aggressive epithelial malignancy in which metabolic alterations have become an important focus of research for targeted and precision therapies. Several clinical studies have reported that elevated lactate concentrations in serum or bile, as well as high LDHA expression in tumor tissue, are associated with metabolic reprogramming, higher pathological grade and stage, and poorer prognosis, suggesting that LDHA-related lactate metabolism may serve as a potential prognostic marker in GBC.

In GBC, experimental data support a prominent glycolytic phenotype. He et al. reported that LDHA is markedly overexpressed in GBC tissues compared with adjacent non-tumor tissues, and that high LDHA expression is an independent predictor of poorer overall survival. In functional assays, RNA interference–mediated knockdown of LDHA or pharmacologic inhibition with the LDHA inhibitor FX11 reduced glycolytic activity, suppressed GBC cell proliferation, invasion and colony formation, promoted apoptosis *in vitro*, and inhibited tumor growth *in vivo*. In the same study, LDHA was identified as a direct target of miR-30d-5p, and loss of miR-30d-5p in GBC tissues was associated with worse prognosis, supporting a role of the miR-30d-5p/LDHA axis in driving aggressive tumor behavior (78).

Metabolomic and epigenetic findings further underscore the importance of lactate-related pathways in biliary tract disease. A proton nuclear magnetic resonance–based serum metabolomics study in patients with chronic cholecystitis identified higher serum lactate and other metabolite alterations compared with controls, and suggested that such metabolic profiles may be relevant to the development of gallbladder disorders, including gallbladder cancer (76). In cholangiocarcinoma, Cai et al. showed that the RNA methyltransferase METTL3 is upregulated in tumour tissue, and that METTL3 overexpression promotes proliferation, migration, invasion, glucose uptake and lactate production by enhancing m6A modification and expression of AKR1B10; METTL3 knockdown had the opposite effects and suppressed tumour growth *in vivo*. Together, these studies indicate that glycolysis- and lactate-associated pathways are important features of biliary tract malignancies and support further evaluation of lactate metabolism–related molecules as candidate biomarkers and experimental therapeutic targets in GBC (75).

#### 4.6.2 Therapeutic targets

GBC is highly aggressive and current systemic therapies provide limited benefit, which has prompted interest in metabolic targeting. Chen et al. showed that miR-139-5p is downregulated in GBC and that low expression is associated with poor prognosis; they further identified pyruvate kinase M2 (PKM2) as a direct target, and restoration of miR-139-5p reduced PKM2 expression, glycolytic flux and tumour growth. These findings indicate that the miR-139-5p/PKM2 axis is an important regulator of glycolysis and malignant behavior in GBC and may offer a basis for metabolism-oriented interventions (74).

Tao et al. reported in experimental GBC models that fasting upregulates the E3 ubiquitin ligase RNF152, which promotes ubiquitination and degradation of the lysosomal protein p18, thereby restraining mTORC1 activity, lowering glycolytic activity and enhancing gemcitabine sensitivity; RNF152 silencing had the opposite effects. This work suggests that the RNF152–p18–mTORC1 pathway links glycolytic reprogramming to drug response in GBC and represents a candidate target for combination strategies with chemotherapy (126).

A recent review by He and colleagues highlighted lactylation as an emerging posttranslational modification that connects the Warburg effect to epigenetic regulation and therapy resistance across multiple cancers. To date, lactylation has not been systematically investigated in GBC, but these insights raise the hypothesis that lactate-driven lactylation might intersect with pathways such as PKM2 or mTORC1 in this disease. Defining such links could help to map metabolic and epigenetic networks and guide future strategies that jointly target glycolysis and lactylation related processes in GBC (52).

### 4.7 Lactylation in other cancer types

In addition to its well-documented role in digestive system cancers, lactylation has emerged as an important epigenetic modification in a variety of other cancer types. The modification of proteins by lactylation affects metabolic reprogramming, immune evasion, and therapeutic resistance in these cancers as well (**Table 4**).

**Table 4:** Therapeutic targets of lactylation modification in various cancer types and their implications.

Cancer type	Lactylation target	Therapeutic target description	Therapeutic implication
Breast Cancer (127, 128)	H3K9la, LDHA	Lactylation promotes proliferation and invasion in breast cancer cells, particularly under hypoxic conditions, with H3K9la and LDHA upregulation enhancing tumor invasiveness and immune evasion.	Targeting LDHA or H3K9 lactylation may reduce tumor growth and improve chemotherapy sensitivity by enhancing immune responses.
Lung Cancer (129)	H3K18la, β-catenin	In lung cancer cells, H3K18 lactylation affects β-catenin transcriptional activity, promoting tumor growth and metastasis.	Inhibiting H3K18 lactylation or β-catenin may reduce tumor invasiveness and improve targeted therapy outcomes.
Prostate Cancer (130)	H4K12la, SHMT2	Lactylation modifies SHMT2, promoting glycolysis and tumor proliferation, while H4K12la plays a crucial role in cancer stem cell characteristics.	Targeting SHMT2 or H4K12 lactylation may suppress glycolysis and enhance chemotherapy/immunotherapy efficacy in prostate cancer.
Ovarian Cancer (131)	MCT1, H3K9la	MCT1 plays a key role in lactate transport in ovarian cancer cells, while H3K9 lactylation activates gene transcription and promotes tumor growth.	Targeting MCT1 and H3K9 lactylation could improve metabolic control and enhance immune recognition and inhibition of tumor growth.
Renal Cancer (132)	PDK1, SHMT2	Lactylation enhances PDK1 activity, supporting glycolysis and tumor cell survival, while SHMT2 lactylation regulates one-carbon metabolism.	Targeting PDK1 and SHMT2 lactylation may inhibit glycolysis and reduce tumor progression in renal cancer.
Melanoma (22)	H3K18la, LDHA, MCT4	Lactylation enhances the expression of LDHA and MCT4 in melanoma cells, promoting lactate metabolism, tumor proliferation, and drug resistance.	Targeting LDHA or MCT4 can inhibit lactate efflux, reducing metabolic adaptation and increasing chemotherapy/immunotherapy sensitivity.

## 5 Conclusions and prospects

This review examines the role of lactylation in gastrointestinal cancers, particularly its pivotal role in tumor progression, metabolic reprogramming, and immune evasion. Lactylation is closely associated with tumor proliferation, migration, and chemotherapy resistance, with the lactylation score demonstrating prognostic value in certain gastrointestinal cancers. Lactylation-related biomarkers hold promise for enhancing early cancer diagnosis and offering new insights for personalized treatment. Despite the significant potential of lactylation in cancer research, the standardization of its detection methods and the clinical application of lactylation-targeted therapies require further exploration. Future research should focus on the role of lactylation in immunotherapy and evaluate the combined application of lactylation modulation with existing treatment regimens to enhance therapeutic efficacy. Overall, lactylation provides a new research direction for the early diagnosis and treatment of gastrointestinal tumors, holding substantial clinical application prospects.
